# Supplementary figures and images for: A voxel-based analysis of cerebral blood flow abnormalities in obsessive-compulsive disorder using pseudo-continuous arterial spin labeling MRI
Source: PLoS One. 2020 Jul 24;15(7):e0236512. doi: 10.1371/journal.pone.0236512 (PMC7380600; doi:10.1371/journal.pone.0236512)

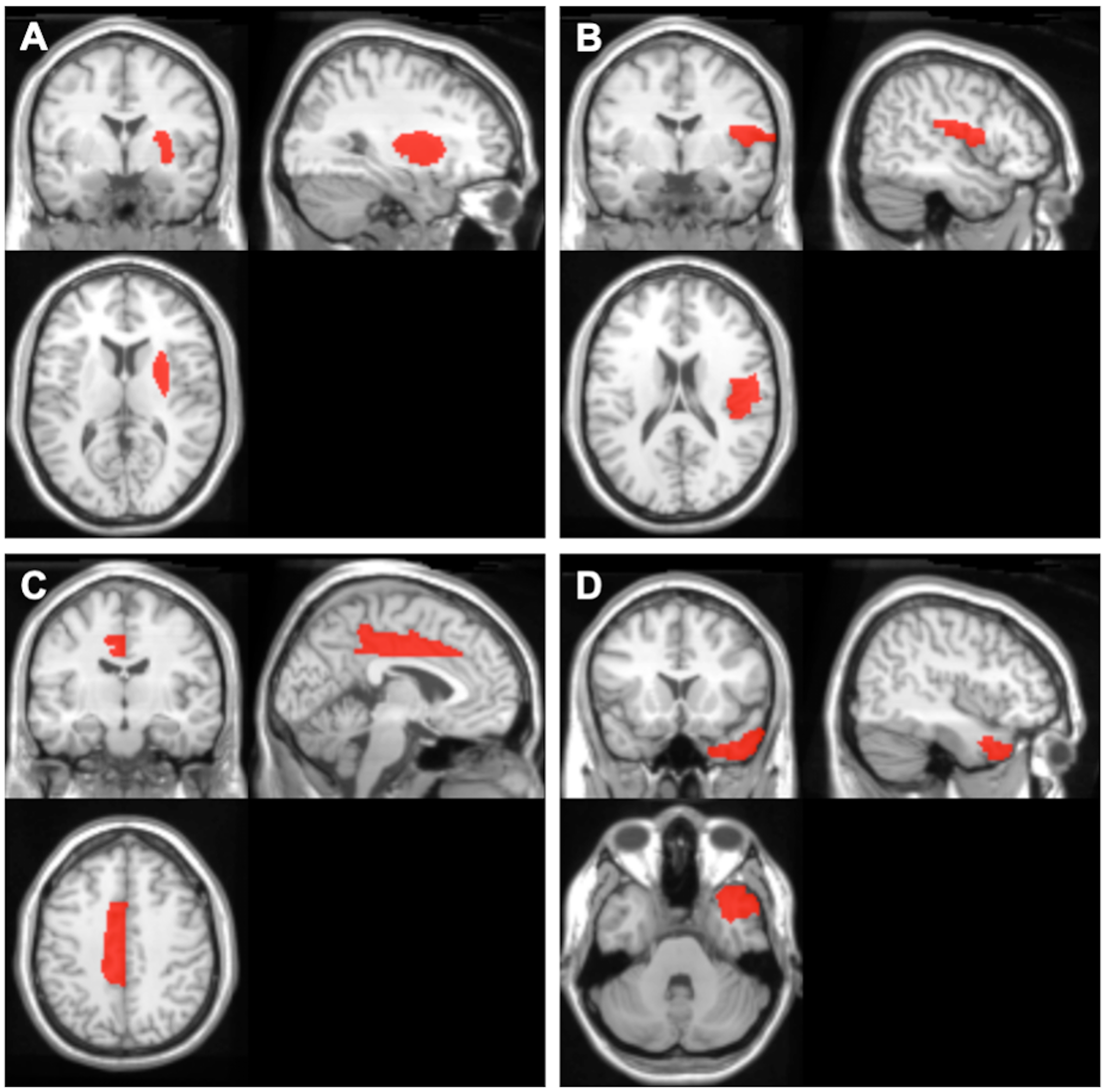

Supplement: S1 Fig — (TIFF) [file pone.0236512.s001.tiff]
