# Supplementary material for: A voxel-based analysis of cerebral blood flow abnormalities in obsessive-compulsive disorder using pseudo-continuous arterial spin labeling MRI
Source: PLoS One. 2020 Jul 24;15(7):e0236512. doi: 10.1371/journal.pone.0236512 (PMC7380600; doi:10.1371/journal.pone.0236512)
Supplement: S4 Appendix — (DOCX) [file pone.0236512.s005.docx]

# S4 Appendix

## Materials and Methods

### Subjects

The subjects were the same as the study population described in the main text: 23 patients with obsessive-compulsive disorder (OCD) (age, 37.2 ± 10.7 years; 9 men and 14 women) and 64 healthy controls (age, 38.3 ± 12.8 years; 27 men and 37 women).

### Image evaluation

A region of interest (ROI)-based analysis was performed focusing on the areas corresponding to the right putamen, the right frontal operculum, the left midcingulate cortex, and the right temporal pole, as the results of the voxel-based analysis demonstrated a significant regional cerebral blood flow (rCBF) reduction in the OCD patients compared to the healthy controls. With the freeware library Anatomical Automatic Labeling (http://marsbar.sourceforge.net) [1], ready-made ROIs for the MNI space consisting of the right putamen, the right Rolandic operculum, the left mid-cingulum, and the right mid-temporal pole were selected (S1 Fig). We used MarsBar software (http://marsbar.sourceforge.net) [2] running on SPM12 to extract the mean rCBF values within the selected ROIs on the preprocessed rCBF maps.

##### **S1 Fig. Ready-made regions of interest (ROIs) for the MNI space selected from the Anatomical Automatic Labeling (AAL) library.** ROIs of (**A**) the right putamen, (**B**) the right Rolandic operculum, (**C**) the left mid-cingulum, and (**D**) the right mid-temporal pole superimposed on the MNI T1 template are shown.


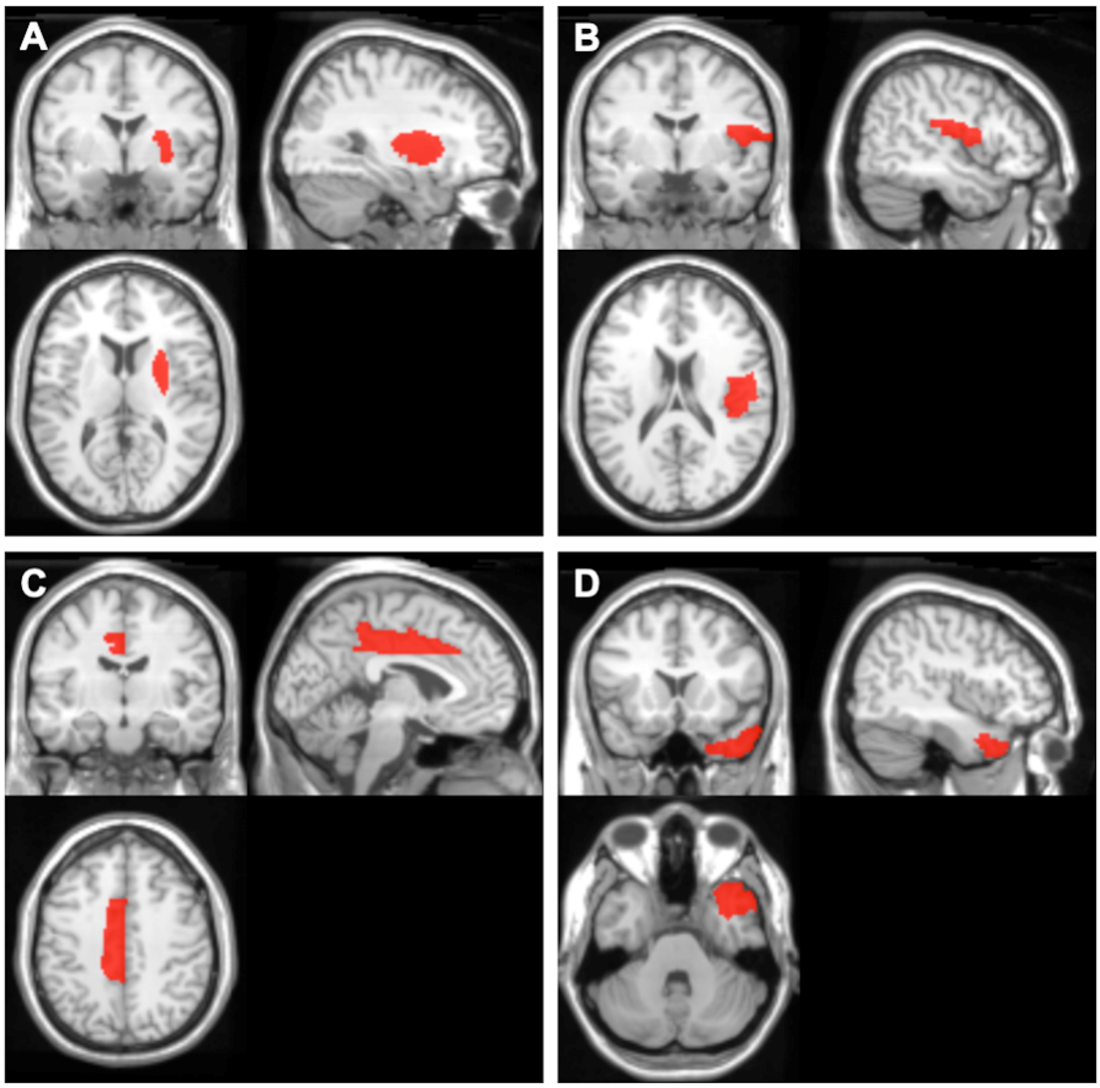


### Statistical analysis

The mean rCBF values within the ROIs were compared between the OCD patients and healthy controls using Student's t-test. P-values <0.05 were accepted as significant. The statistical analyses were performed using JMP Pro 14.2.0 (SAS Institute, Cary, NC).

## Results

S1 Table shows the mean rCBF values within the ROIs of the OCD patients and healthy controls. The mean rCBF within the right putamen and the right mid-temporal pole were significantly lower in the OCD patients than in the healthy controls. There were no significant differences in mean rCBF within the left mid-cingulum or the right Rolandic operculum between the OCD and control groups.

| **S1 Table. Mean rCBF values within the ROIs in the OCD patients and healthy controls.** | | | |
| --- | --- | --- | --- |
|  | **OCD**  **(n=23)** | **Healthy controls**  **(n=64)** | **p-value** |
| R. putamen | 36.3 ± 5.2 | 39.3 ± 5.8 | 0.0333* |
| R. Rolandic operculum | 42.3 ± 6.3 | 45.0 ± 7.0 | 0.1033 |
| L. mid-cingulum | 45.5 ± 6.9 | 47.9 ± 7.9 | 0.2047 |
| R. mid-temporal pole | 30.6 ± 5.4 | 46.0 ± 7.2 | <0.1111* |

Data are mean ± SD, ml/min/100 g. *p <0.05. rCBF, regional cerebral blood flow; OCD, obsessive-compulsive disorder.

## References

###### 1. Tzourio-Mazoyer N, Landeau B, Papathanassiou D, Crivello F, Etard O, Delcroix N, et al. Automated anatomical labeling of activations in SPM using a macroscopic anatomical parcellation of the MNI MRI single-subject brain. NeuroImage. 2002;15(1):273-89.

###### 2. Brett M, Anton JL, Valabrgue R, Poline J-B. Region of interest analysis using an SPM toolbox. Presented at the 8th International Conference on Functional Mapping of the Human Brain, June 2-6, 2002, Sendai, Japan. NeuroImage. 2002;13:210-7.
