# Supplementary material for: A voxel-based analysis of cerebral blood flow abnormalities in obsessive-compulsive disorder using pseudo-continuous arterial spin labeling MRI
Source: PLoS One. 2020 Jul 24;15(7):e0236512. doi: 10.1371/journal.pone.0236512 (PMC7380600; doi:10.1371/journal.pone.0236512)
Supplement: Attachment — Submitted filename: Response to Reviewers.docx [file pone.0236512.s007.docx]

Reviewer #1:

Major comments:

1, In the inclusion criteria of OCD patients, the authors did not mention whether the patients have the comorbidity of depression and anxiety symptoms, as comorbidity issue is very common in the clinically psychiatric diagnosis. The author should at least address this point. If yes, whether this comorbidity could have an impact on the results.

**Response:** In this study, we excluded OCD patients with a comorbid axis I diagnosis (including mood and anxiety disorders) by using the structured clinical interview for the DSM-IV axis I disorders, patient edition (p.8, lines 99-102). Thus, none of the OCD patients in this study had any comorbid axis I disorders.

2, The authors reported the results of both nonparametric and parametric tests. I think nonparametric results are good enough to illustrate the main findings of this study and more strict to identify the abnormal regions in OCD in this paper. So, I would suggest to remove the results of parametric results.

**Response:** We have removed the results for the parametric analyses as suggested. All analyses including the comparison of rCBF and gray matter volume between the OCD patients and healthy controls and the regression analysis within the OCD patients used the nonparametric test. The results obtained with the nonparametric test were the same as those obtained with the parametric test.

3, In the introduction, the author mentioned higher signal-to-noise ratio in the pCASL, which could be helpful to sensitively detect the brain abnormalities in OCD. Moreover, they found lower rCBF in the regions of the temporal pole and frontal operculum, which is in or very close to the regions with lower signal-to-noise ratio in the neuroimaging correction. Could the authors test the effect of SNR among these regions showing between-group differences?

**Response:** In general, ASL using EPI inevitably suffers from susceptibility artifacts in mesial temporal regions due to the proximity of the skull base and air-containing structures (Petcharunpaisan et al., World J Radiol 2010;2). Distortions of raw pCASL images in regions of high magnetic field susceptibility may lead to a low signal-to-noise ratio (SNR) to some extent; we have therefore measured SNRs within the circular region-of-interest (ROI) placed on the right temporal pole and the right frontal operculum on the label images of the pCASL in all subjects. The SNR was also measured in the right cerebellar hemisphere as a reference.

We calculated the SNRs by using the following formula (Yu et al., BMC Medical Imaging 2018;18):

$$SNR=0.655 \times{\mu_{tissue}}/{\sigma_{air}}$$

where $\mu_{tissue}$ is the mean signal intensity within the ROI positioned on each area of the brain, and $\sigma_{air}$ is the standard deviation of the signal intensity within the ROI placed on the air.

As a result, the SNR in the right temporal pole was high enough, and there was no major difference in mean SNR between the right temporal pole and the right cerebellar hemisphere (175.8 ± 104.3 vs. 214.0 ± 124.5).

In addition, as we did not directly analyze raw pCASL images but rather focused on the between-group differences in quantitative rCBF maps, we think that the SNR of the raw pCASL image doesn’t critically affect the final results. However, since the SNR of the right temporal lobe was a little lower in fact, we have added this issue to the limitation section of the Discussion (p. 23-24, line 348-354).

The SNR of the right frontal operculum was also high enough and not lower than that of the right cerebellar hemisphere (358.8 ± 260.0 vs. 214.0 ± 124.5).

4, In the discussion, the authors could add more discussion about the relationship between decreased rCBF and its associated disrupted pathophysiology of OCD. In other words, why does OCD show decreases of rCBF, rather than increased rCBF? What does lower rCBF imply in the links with the symptomatology of OCD?

**Response:** Considering the neurovascular coupling theory, the reduction in rCBF shown in the present study might reflect functional decline of the corresponding areas. We have clarified this point throughout the Discussion section and more thoroughly discussed the relationships between the functional decline of the areas detected in the present study and the symptomatology of OCD, referencing previous functional MRI studies (p.17-20, 252-299).

Minor comments:

1, In the abstract, the authors said the alterations of cerebral perfusion may precede structrual changes in OCD, which does not make sense and is not rigorous. The authors did not have longitudinal results to support this point. It's very difficult to make a conclusion of the order of functional and structural changes for the current results. The authors could just say there is no structural changes in OCD while physiological disruption occurs in OCD patients. Thus, I would suggest to remove this kind of description in the manuscript.

**Response:** We have removed the description about the order of functional and structural changes. Instead, we concluded as follows: it was possible that the ASL could sensitively detect physiologically impaired areas without structural abnormalities even in the small sample size (p.21, line 315-317).

2, In the Methods, the authors did not mention the resample of neuroimaging. Resample to 3*3*3?

**Response:** Thank you for pointing this out. In the present study, the rCBF maps were first resampled to 1×1×1 mm. Specifically, the M0 images are co-registered and resampled to the 3D T1WIs with 1×1×1 mm resolution; then, the same transformations were applied to the raw rCBF maps. After the normalization to the brain template and smoothing, the resolution was finally changed to 2×2×2 mm. We have added a new Fig 2 which illustrates the details of the preprocessing steps applied to the pCASL maps.

3, Even though the authors regressed out total brain volume in the between-group comparisons of rCBF, they should report the results of between-group comparison of total brain volume before they do the VBM analysis. Because sometimes structural changes did not meet the multiple testing in voxel-level analysis, while it could have subtle changes in the global or regional level.

**Response:** Thank you for this suggestion. We have compared the total brain volume between the OCD patients and healthy controls and confirmed that there was no significant difference. We have added this result to Table 1. In addition, detailed subjects’ information including total brain volume has been added as supplemental data (S1 and S2 Appendices).

4, In the Methods, the authors run the nonparametric analysis with 5000-time permutation. Why not choose 10000 permutations, which is more commonly used.

**Response:** We have re-calculated all nonparametric analyses with 10,000-time permutations. The main findings are identical. We have replaced the previous 5,000-permuations results with the new results with 10,000-time permutations.

Reviewer #2:

The data is not clearly explained and published, which may violate the PLOS Data policy. It's suggested to give a location of the data used and made it public available. Specifically, the statistic analysis part doesn't give a detail about data points, there is only limited summary and overview.

**Response:** We have added the subjects' detailed information including individual age, gender, education years, total brain volume, illness duration, and Y-BOCS score as supplemental data (S1 and S2 Appendices). The raw results of the rCBF comparison between the OCD patients and healthy controls acquired by using SnPM13 were also added as S3 Appendix.

Regarding the statistical analysis section, we have added descriptions of the analytical methods, tools and parameters in as much detail as possible (p.13-14, line 179-198).

The experiment is a little bit weak to support the effectiveness of proposed approach. It's suggested to compare the results with other baseline approaches (for example, ROI-based approach). Such comparison will make it more clear to readers and highlights the improvement over other existing methods.

**Response:** We have performed an ROI-based analysis in addition to the main voxel-based analysis in order to compare the analytical methods. The ROI-based analysis was performed focusing on the areas corresponding to the right putamen, the right frontal operculum, the left midcingulate cortex, and the right temporal pole, which demonstrated significant rCBF reductions in the OCD patients compared to the healthy controls in the voxel-based analysis. The detailed information of the additional ROI-based analysis is provided as Appendix S4.

The ROI-based analysis detected a reduction of the rCBF in the areas corresponding to the right putamen and right temporal pole; however, it failed to detect an rCBF reduction in the right frontal operculum or the left midcingulate cortex (p.16, line 231-237). This might be because the ROIs gathered together large heterogeneous subregions with separate functional roles, resulting in the missing of small foci of decreased rCBF. Thus, it seems that the voxel-based approach has an advantage over the ROI-based approach in detecting smaller areas with rCBF abnormalities. We have added this description to the Discussion (p.22-23, line 327-335).
